# Is Sudden Hearing Loss Associated with Atherosclerosis?

**Published:** 2016-05

**Authors:** Mohsen Rajati, Mahmoud Reza Azarpajooh, Mohsen Mouhebati, Mostafa Nasrollahi, Maryam Salehi, Ehsan Khadivi, Navid Nourizadeh, Firoozeh Hashemi, Mehdi Bakhshaee

**Affiliations:** 1 *Sinus and Surgical Endoscopic Research Center, Ghaem Hospital, Faculty of Medicine, Mashhad University of Medical Sciences, Mashhad, Iran. *; 2 *Department of Neurology, Ghaem Hospital, Faculty of Medicine, Mashhad University of Medical Sciences, Mashhad, Iran.*; 3 *Department of Cardiology, Ghaem Hospital, Faculty of Medicine, Mashhad University of Medical Sciences, Mashhad, Iran.*; 4 *Department of Otorhinolaryngology, Ghaem Hospital, Faculty of Medicine, Mashhad University of Medical Sciences, Mashhad, Iran. *; 5 *Department of Community Medicine, Faculty of Medicine, Mashhad University of Medical Sciences, Mashhad, Iran. *

**Keywords:** Atherosclerosis, risk factors, Carotid Intima-Media Thickness, Doppler C-reactive protein, Ultrasonography, Sudden sensorineural hearing loss

## Abstract

**Introduction:**

Sudden sensorineural hearing-loss (SSNHL) patients constitute approximately 2–3% of referrals to ear, nose and throat (ENT) clinics. Several predisposing factors have been proposed for this condition; one of which is vascular disorders and perfusion compromise. In this research the atherosclerotic changes and their known risk factors are studied in SSNHL patients.

**Materials and Methods:**

Thirty SSNHL patients and 30 controls were evaluated with regard to cardiovascular risks including history, heart examination, blood pressure, body mass index, waist circumference, electrocardiogram, blood sugar, triglycerides, cholesterol, high-sensitivity C-reactive protein (HSCRP); also, carotid artery color Doppler study was undertaken to measure intima media thickness(IMT).

**Results:**

IMT and HSCRP showed an increased risk in the case group compared with the controls (P= 0.005 & P=0.001). However, waist circumference, history of smoking, fasting blood sugar, lipid profile, and electrocardiogram revealed no significant difference between the two groups. Interestingly, blood pressure and body mass index were higher in the controls in this study.

**Conclusion:**

Sudden sensorineural hearing loss may be associated with subclinical atherosclerosis.

## Introduction

Sudden sensorineural hearing loss (SSNHL) is defined as loss of hearing of over 30 dB in three sequential frequencies occurring within 3 days (1,2). The annual incidence of SSNHL has been reported to be 10 in 100,000 people (3,4).

 Different mechanisms are thought to lead to this condition including viral infections, vascular obstruction, autoimmunity, and rupture of the endocochlear membranes (5-7). In 10–15% of patients the cause may be known only after a comprehensive clinical and paraclinical evaluation. The known etiologies include posterior fossa tumor, trauma, Ménière's disease, and ototoxicity, for example. Other cases remain idiopathic and are almost always unilateral (8,9).

On the other hand, there are some known risk factors for atherosclerosis including age, gender, obesity, blood pressure, lipid profile, high-sensitivity C-reactive protein (HSCRP), and smoking (10-12). 

Atherosclerotic changes in the carotid artery are also an important indicator of risk of stroke or myocardial infarction (MI)(13).

Because vascular factors have been proposed to be a possible underlying mechanism of this condition, we examined atherosclerosis risk factors in SSNHL patients in this study. 

## Materials and Methods

In this cross-sectional study, patients with sudden sensorineural hearing loss admitted at Ghaem Hospital, a tertiary teaching medical center, between September 2011 and September 2012 were selected. These patients were visited in the otolaryngology clinics because of their hearing loss within the first three days of the beginning of their symptoms. The control group did not suffer from any known cardiovascular or neurological conditions or hearing problems. They were selected from a large healthy community and underwent a thorough history, physical examination and doppler exam of upper neck on an outpatient basis. They were matched in terms of age, gender, and residence (the area they lived) on a person-to-person basis. All patients went through a comprehensive evaluation for possible etiologies such as brain tumors and infectious diseases as well as rheumatologic, autoimmune, traumatic and hematologic causes; and those with known etiologies (5 cases of acoustic schwannoma, head trauma and ototoxicity) were excluded from the study.

 All those in the study and the control groups were taken complete history (including smoking history by pack years unit), beside examination from the point of clinical as well as microscopic evaluation by an ear, nose, and throat (ENT) specialist, and also underwent audiometry. Then, they were examined by a cardiologist and underwent a history and physical examination including body mass index (BMI), waist circumference and blood pressure assessment (Multiple measurements at upper extremity with at least half hour intervals). Hypertension defined by blood pressure more than 140/90 mmHg. An electrocardiogram (ECG) was also performed.

Next, all cases and controls were examined by a neurologist and given doppler sonography of the upper neck blood vessels, using a Medison instrument (SONOACE 8000 EX). In order to measure the intima media thickness (IMT), proper magnification on the carotid artery wall was first obtained; then, in the area of the carotid bifurcation between the distal part of the common carotid artery and the proximal part of the internal carotid, IMT was measured on both sides using an automated measurement mode.

Serum tests, including high-density lipoprotein (HDL), low-density lipoprotein (LDL), total cholesterol, triglycerides (TG), fasting blood sugar (FBS), and high-sensitivity C-reactive protein (HSCRP) were performed on all patients, just when they were admitted(before initiation of the medications such as corticosteroid)and the controls.

***Statistical analysis***

All results were analyzed using SPSS, Absolute numbers and percentages were computed to describe data. Data were expressed as mean ± SD for continuous variables Categorical variables were compared between two groups using the chi square test and the mean of two independent groups were compared using Independent T test. For adjusting the confounders, variables were entered into the binary logistic regression model and OR (Odds Ratio) with 95% confidence intervals (CI) calculated. A P-value less than 0.05 were considered as statistically significant.

This study was approved by the Ethics Committee of Mashhad University of Medical Sciences.

## Results

This study was performed in 30 patients suffering from SSNHL compared with 30 non-SSNHL controls. The mean age of the study group was 45±12.7 years (range, 21–65). There were 15 male and 15 female patients. The control group consisted of 13 men and 17 women with a mean age of 45±11.8 years. Sixteen (53.3%) patients had a right-ear involvement and 14 (46.7%) a left-ear involvement. There was no significant difference between the study and the control groups in terms of age (P= 0.29) and gender (P=0.29).

The average speech reception threshold in the case group (the worse ear) was 86.17±15.51 db (range, 50–110 db) and in the control group was9.33±4.49 (range, 5–20 db) (the average of both ears).

Mean BMI was 25.32 ± 4.4 in the case group and 26.49 ±4.9 in the controls, which were not significantly different (P = 0.40); but there was no significant difference in terms of waist circumference.

 Smoking frequency was 16.7% in the study group and 10% in the control group, which showed no statistically significant difference (P=0.93). Hypertension was seen in 7.16% of the study group and 40% of the control group, which was a statistically significant difference (P= 0.045).

 Mean HSCRP levels were markedly higher in the study group than in the control group, and blood sugar and lipid levels, although higher in the study group than in the control group, showed no statistically significant difference (Table. 1).

**Table 1:** The Result of blood test

**Serum item**	**Cases**	**Controls**	**P Value**
HSCRP (high-sensitivity C-reactive protein)	6.98± 6.67 mg/L	2.67 ± 2.8 mg/L	0.001
FBS (fasting blood sugar)	105.3 ± 33.5 mg/dl	100.7 ± 39 mg/dl	0.63
Total Cholesterol	207.8 ± 50.8 mg/dl	196.6 ± 46.7 mg/dl	0.38
Triglycerides	124.2 ± 67.8 mg/dl	131.1 ± 67.3 mg/dl	0.69
HDL (high-density lipoproteins)	51.9 ± 9.6 mg/dl	47.5 ± 13.5 mg/dl	0.16
LDL (low-density lipoproteins)	135.4 ± 37.3 mg/dl	124.6 ± 38.6 mg/dl	0.28

The results of the Doppler study showed that the mean IMT in the right carotid bulb, the right internal carotid, and the left internal carotid (Bulb-R, ICA-R, and ICA-L) were significantly higher in the study group than in the control group (Table. 2).

After adjusting hypertension as a confounding factor among two groups by logistic regression analysis we found that increases in the IMT in Bulb-R(OR=1.101, 95% CI:1.03-1.18)., ICA-R(OR=1.080, 95% CI:1.02-1.14), ICA-L(OR=1.064, 95% CI:1.00-1.23) and bulb-L(OR=1.05, 95% CI:1.00-1.10) are the independent risk factors for sudden hearing loss.

**Table 2:** The result of Intima Media Thickness measurement (mm) with Doppler Sonography in various locations; CCA: common carotid artery, BULB: carotid bulb, ICA: internal carotid artery, R: right, L: left

**Location**	**Cases N=30**	**Controls N=30**	**P-value**
CCA-R	0.49± 0.11	0.49± 0.1	0.9
BULB-R	0.54 ± 0.15	0.44 ± 0.08	0.002
ICA-R	0.52 ± 0.12	0.43± 0.1	0.005
CCA-L	0.5 ± 0.15	0.51 ± 0.14	0.83
BULB-L	0.54 ± 0.16	0.47± 0.11	0.08
ICA-L	0.51 ± 0.15	0.44 ± 0.1	0.04

We also considered the cutoff point of 0.6 mm for intima thickness to compare cases and controls. After hypertension as a confounding factor was entered into the model separately for each location , only the odds ratio for IMT more than 0.6 mm in the right carotid bulb was statistically significant (OR=7.50, 95% CI:1.71-32.54). 

Cardiovascular and neurological physical examinations in all the subjects were normal, while ECGs showed old ischemic changes in two individuals (one in each group). 

## Discussion

SSNHL is one of the most important ENT conditions, manifesting itself as sudden loss of hearing, sometimes with vertigo and tinnitus. SSNHL patients tend to range across all age groups. Various background factors seem to play an etiopathogenic role, including viral, vascular, and immunologic etiologies, but as the actual cause is hard to identify in most cases, the condition is considered idiopathic(1). Due to environmental factors and life stresses, the incidence of SSNHL seems to be on the rise. 

Although when it is treated quickly, there is probably a good chance of some recovery, there is a need for further investigation into the pathogenesis of this condition in view of its morbidity.

 Several possible hypotheses have been suggested to explain the vascular involvement, including sudden nature of the onset of the disease (similar to cardiac or cerebral vascular accidents); histopathological changes due to vascular obstruction (in guinea pigs, the obstruction of labyrinthine vessels leads to a decline in spiral ganglion cells, mild to moderate damage to the organ of Corti, and intracochlear fibrosis)(1); the fact that cochlea depends on a single terminal posterior cerebral blood supply(6); spinal cord manipulation, potentially harming vertebrobasilar vessels, has been shown to cause SSNHL(7). Blocking sympathetic ganglia in the neck has been recognized as an effective treatment through a vasodilation mechanism(14). Also, there are several recent studies showing that cardiovascular risk factors (smoking, increased alcohol consumption) may be associated with a higher risk of developing SSNHL(15). Factor V Leiden and MTHFR gene polymorphisms were found to occur more frequently in patients with SSNHL in several studies (15).

On the other hand, atherosclerosis is a pathologic condition based on inflammatory vascular changes throughout the body. There are several traditional risk factors like diabetes mellitus, hypertension, smoking and dyslipidemia that cause atherosclerosis. Some predisposing factors like aging and family history of premature coronary artery diseases (CAD) may accelerate these changes. Based on paraclinical data, HSCRP is a known predictor of atherosclerosis and acute coronary events. The intima media thickness (IMT) is also a helpful marker based on carotid intimal thickness to predict atherosclerotic changes throughout the body.

 Quantitative assessment of atherosclerosis was performed by Eugene in the early 1980s(16). Pignoli (1984 and 1986) went on to describe the IMT and showed how it matched pathological samples(17). Since that time, several studies have shown IMT as a reliable criterion for the assessment of atherosclerosis(18). Thus, any change or increase in IMT can indicate the beginning of this process throughout the body.

 This technique provides information about atherosclerotic changes which cannot be obtained through angiography or MRI. B-Mode sonography has shown that an increase in IMT directly correlates with risk of MI and stroke, especially in older patients without a history of cardiovascular conditions (13).

IMT measurements at different sites of the carotid arteries have been performed in different groups, and have shown that the common carotid artery IMT (CCA IMT) is a good predictor of stroke, and that the internal carotid artery IMT (ICA IMT) is a good predictor of MI(16). Epidemiological evidence shows a clear increase in the incidence of cardiovascular events in individuals with an IMTs 1 mm(19).

In view of the mean IMT in this study group, which differed significantly from that of the control group, atherosclerosis may be considered an important factor in these patients; however, IMT values were lower than the risk threshold of 1 mm. On the other hand, as atherosclerosis is a whole-body phenomenon, changes of as little as 0.1 mm in small vessels such as the labyrinthine artery (which is only 0.5 mm in diameter) can deleteriously influence the blood flow (20). Also, Atherosclerosis is known to predispose clot formation, which may get free and circulate into the terminal labyrinthine artery or its branches; this can end up in SSNHL.

Ciccone et al studied flow-mediated dilation (FMD) of the brachial artery, which is an early index of subclinical atherosclerosis, and found it to be significantly lower in patients experiencing sudden deafness; in their study IMT values of carotid artery were also within the normal range (21).

Inflammatory biomarkers are believed to have important role in the pathophysiology of SSNHL(22). Recent studies have shown that CRP is also formed in the intimal layer of atherosclerotic vessels. Thus, apart from being an inflammatory marker, CRP can also damage vessels through various mechanisms(12).

The American Heart Association and the US Centers for Disease Control and Prevention have recently offered a strategy for the practical use of CRP; CRP levels of less than 1 mg/l indicate low cardiovascular risk, 1–3 mg/l moderate risk and over 3 mg/l high risk (23,24). In current study, the HSCRP was also significantly higher than in the control group (the mean HSCRP was 2.67 mg/l in the control group, and 6.98 mg/l in the study group), which can at least suggest subclinical atherosclerosis. Recent studies have shown that patients with high CRP levels (without other risk factors) will benefit from treatment with statins (11). 

In the end the authors would like to emphasize that, as mentioned in the methodology section, the controls of this study were not truly *randomly* chosen individuals, rather biased toward the atherosclerosis risk factors. And this was due to the limitation of doing upper neck doppler exam in all the controls. This selection bias accounts for the high prevalence of hypertension in the controls. However, the results in a non-biased control group are expected to emphasize our findings more markedly. Considering the role of inflammation in atherosclerosis, the authors believe the pathophysiology of SSNHL may be closely connected to the vascular background of the inner ear; this impression certainly needs further investigations to clarify the details of this relationship.

## Conclusion

There are several pieces of evidence indicating that vascular atherosclerotic changes may be important in the pathophysiology of SSNHL, at least in a number of patients. HSCRP and IMT are the new findings proposed in this paper. 
